# Mdivi-1 Protects Adult Rat Hippocampal Neural Stem Cells against Palmitate-Induced Oxidative Stress and Apoptosis

**DOI:** 10.3390/ijms18091947

**Published:** 2017-09-11

**Authors:** Sehee Kim, Chanyang Kim, Seungjoon Park

**Affiliations:** 1 Department of Biomedical Science, Graduate School, Kyung Hee University, Seoul 02447, Korea; shysl@hanmail.net (S.K.); praise1107@naver.com (C.K.); 2 Department of Pharmacology and Medical Research Center for Bioreaction to ROS and Biomedical Science Institute, School of Medicine, Kyung Hee University, Seoul 02447, Korea

**Keywords:** mdivi-1, mitochondrial fission, oxidative stress, apoptosis, palmitate, lipotoxicity, Drp1

## Abstract

Palmitate concentrations in type 2 diabetic patients are higher than in healthy subjects. The prolonged elevation of plasma palmitate levels induces oxidative stress and mitochondrial dysfunction in neuronal cells. In this study, we examined the role of mdivi-1, a selective inhibitor of mitochondrial fission protein dynamin-regulated protein 1 (Drp1), on the survival of cultured hippocampal neural stem cells (NSCs) exposed to high palmitate. Treatment of hippocampal NSCs with mdivi-1 attenuated palmitate-induced increase in cell death and apoptosis. Palmitate exposure significantly increased Drp1 protein levels, which were prevented by pretreatment of cells with mdivi-1. We found that cytosolic Drp1 was translocated to the mitochondria when cells were exposed to palmitate. In contrast, palmitate-induced translocation of Drp1 was inhibited by mdivi-1 treatment. We also investigated mdivi-1 regulation of apoptosis at the mitochondrial level. Mdivi-1 rescued cells from palmitate-induced lipotoxicity by suppressing intracellular and mitochondrial reactive oxygen species production and stabilizing mitochondrial transmembrane potential. Mdivi-1-treated cells showed an increased Bcl-2/Bax ratio, prevention of cytochrome c release, and inhibition of caspase-3 activation. Our data suggest that mdivi-1 protects hippocampal NSCs against lipotoxicity-associated oxidative stress by preserving mitochondrial integrity and inhibiting mitochondrial apoptotic cascades.

## 1. Introduction

Diabetes is a metabolic disorder characterized by chronic hyperglycemia, which is harmful to brain function. The most common complications in the diabetic brain involve cognitive impairment and depression [1]. It has been reported that adult neurogenesis is decreased in type 1 [2,3] and type 2 diabetic rats [4,5] in the hippocampal dentate gyrus subgranular zone, one of the two places where neurogenesis continues throughout life [6]. Although the precise mechanisms of decreased adult hippocampal neurogenesis in diabetes have not been clarified yet, previous studies revealed that increased reactive oxygen species (ROS) levels and mitochondrial dysfunction are associated with this phenomenon [7]. Given that hippocampal-dependent cognitive performance can be improved when adult neurogenesis in the hippocampus is enhanced [8,9], therapeutic substances targeting the protection of hippocampal neurogenesis in the diabetic brain may be probable candidates for the treatment of diabetes-mediated cognitive impairment. For instance, previous studies suggested that ghrelin gene products—acylated ghrelin, unacylated ghrelin and obestatin—promote hippocampal neurogenesis [10,11,12,13,14] and rescue adult rat hippocampal neural stem cells (NSCs) from glucotoxicity [15].

Sustained elevation of saturated free fatty acids (FFAs), such as palmitate, resulting in lipotoxicity is an important factor in the onset of insulin resistance, type 2 diabetes mellitus and cardiovascular disease [16,17]. Non-adipose cells, such as skeletal muscle, liver, pancreas and heart, are vulnerable to lipotoxicity [18]. In addition, increased levels of FFAs are cytotoxic to neuronal cells because FFAs can cross the blood–brain barrier and access most areas of the brain [19]. Various neuronal cells exposed to high levels of palmitate show serious cellular dysfunction and death [20,21,22,23,24], which lead to impairment of learning and memory abilities. Considering that elevated levels of circulating FFAs are implicated in diabetic neuropathy, it is plausible that the protection of neuronal cells through the inhibition of lipotoxicity may attenuate the progression of neuropathy in patients with diabetes. This may be more important in obese type 2 diabetes mellitus patients as obese individuals have high concentrations of circulating FFAs [16,17]. Palmitate rate of appearance is 1.5- and 3-fold higher in type 2 diabetic subjects during nocturnal and postprandial states, respectively, than in control subjects [25] and it has been reported that circulating palmitate levels are approximately 75 μM in rats [26].

Mitochondria are not static but highly dynamic intracellular organelles that continuously change their shape through fission and fusion. These processes are essential in the maintenance of mitochondrial integrity and quantity and the regulation of critical mitochondrial functions, such as cellular respiration, membrane potential, apoptosis, calcium homeostasis, and ROS generation [27]. Mitochondrial fission and fusion are precisely regulated by the mitochondrial fission proteins dynamin-regulated protein 1 (Drp1) and fission protein 1 and by the mitochondrial fusion proteins mitofusin 1 and 2 and optic atrophy 1 [27]. The opposing actions of fission and fusion proteins contribute to the maintenance of the morphology of the mitochondrial networks. Mitochondrial fusion produces larger and highly interconnected networks of mitochondria, whereas small fragmented organelles are generated through fission. Among these mitochondrial fission and fusion proteins, special attention has been paid to Drp1 because of its role in mitochondrial phenotypes [28]. Mitochondrial morphology needs to be finely regulated in a living cell to maintain cellular function.

Disruption between the balance of mitochondrial fission and fusion is associated with increased susceptibility to apoptotic cell death in various cell types. For instance, overexpression of Drp1 in H9c2 myocytes induces mitochondrial fragmentation with mitochondrial inner transmembrane potential (Δψ_M_) depolarization and the evidence of insulin resistance [29]. The excess palmitate-induced mitochondrial fragmentation and fission were observed in differentiated C2C12 muscle cells [30] and cultured cardiomyocytes [31]. On the other hand, mitochondrial dysfunction and insulin resistance induced by palmitate were attenuated by genetic or pharmacological inhibition of Drp1 [30]. Furthermore, mdivi-1, the first selective inhibitor of the mitochondrial fission protein Drp1 [32], has been suggested to have a protective effect on hippocampal neurons exposed to ischemia/reperfusion [33,34,35] and kainic acid-induced injury [36] via suppressing oxidative stress and improving mitochondrial function. Taken together, these findings suggest that mdivi-1 may improve mitochondrial dynamics and function of adult rat hippocampal NSCs against palmitate insult. At present, no data are available on the effect of mdivi-1 in hippocampal NSCs exposed to high palmitate. In this study, we investigated the role of mdivi-1 on the survival of hippocampal NSCs exposed to palmitate. Specifically, we assessed changes in apoptosis, Drp1 expression, ROS production, Δψ_M_, cytochrome c release, caspase-3 activation, and the Bcl-2 family of proteins.

## 2. Results

### 2.1. Mdivi-1 Protects Hippocampal NSCs against Palmitate Insult

**Figure 1:**
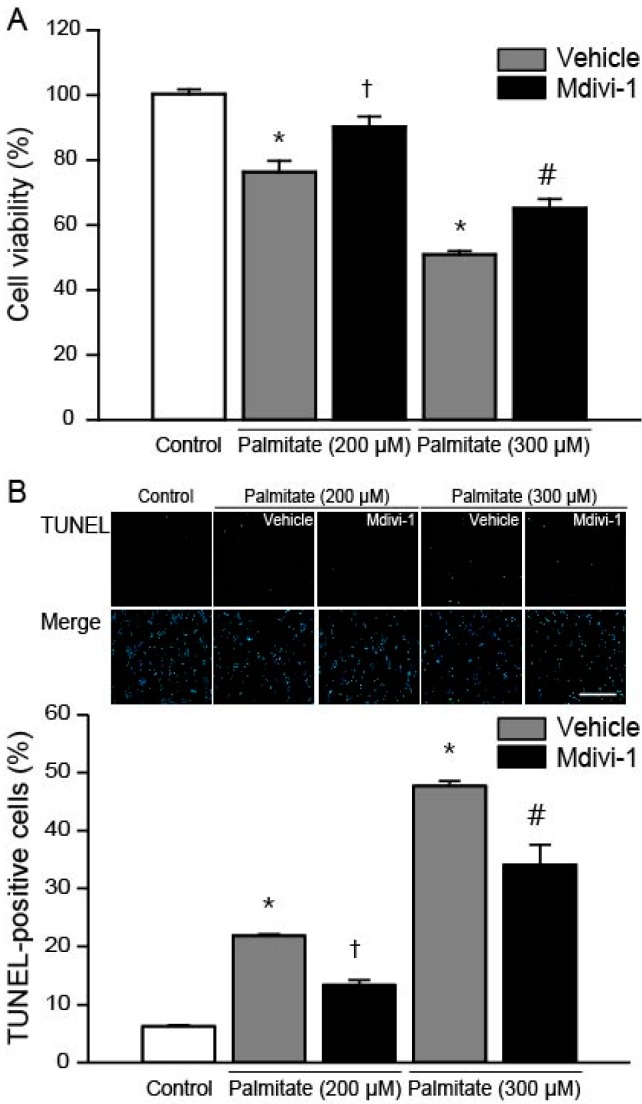
Mdivi-1 protects hippocampal NSCs against palmitate insult. Cells were pretreated with dimethyl sulfoxide (DMSO) vehicle or mdivi-1 (20 μM) for 2 h and then exposed to 0.5% bovine serum albumin (BSA) vehicle or palmitate (200 and 300 μM) for 24 h. (**A**) Cell viability was measured by the 3-(4,5-dimethylthiazol-2-yl)-2,5-diphenyltetrazolium bromide (MTT) assay; (**B**) Apoptosis was assessed by terminal deoxynucleotidyl transferase dUTP nick end labeling (TUNEL) staining. Nuclei were counterstained with 4′,6-diamidino-2-phenylindole (DAPI) and images were merged with the TUNEL stain (Merge). Scale bar = 200 μm. Values are the mean ± standard error of the mean (SEM). of four separate experiments performed in triplicate. * *p* < 0.05 vs. DMSO-treated control; † *p* < 0.05 vs. 200 μM palmitate; # *p* < 0.05 vs. 300 μM palmitate.

To investigate whether mdivi-1 may act as a survival factor for hippocampal NSCs, we assayed the effect of mdivi-1 on cell death induced by palmitate insult. As shown in Figure 1A, palmitate-induced cell death was partially, but significantly, reduced by a 2-h pretreatment with mdivi-1 (20 μM). Next, we investigated the effect of palmitate on apoptosis in hippocampal NSCs. The percentage of TUNEL-positive cells compared with vehicle-treated control significantly increased to 22% and 48% when cells were exposed for 24 h to 200 μM and 300 μM palmitate, respectively (Figure 1B). However, palmitate-induced apoptosis was significantly attenuated by pretreatment with mdivi-1 (Figure 1B). In addition, it was reported that mdivi-1 alone treatment did not produce a statistically significant effect on cell viability and apoptosis [37].

### 2.2. Mdivi-1 Inhibits Palmitate-Induced Drp1 Expression and Mitochondrial Translocation

**Figure 2:**
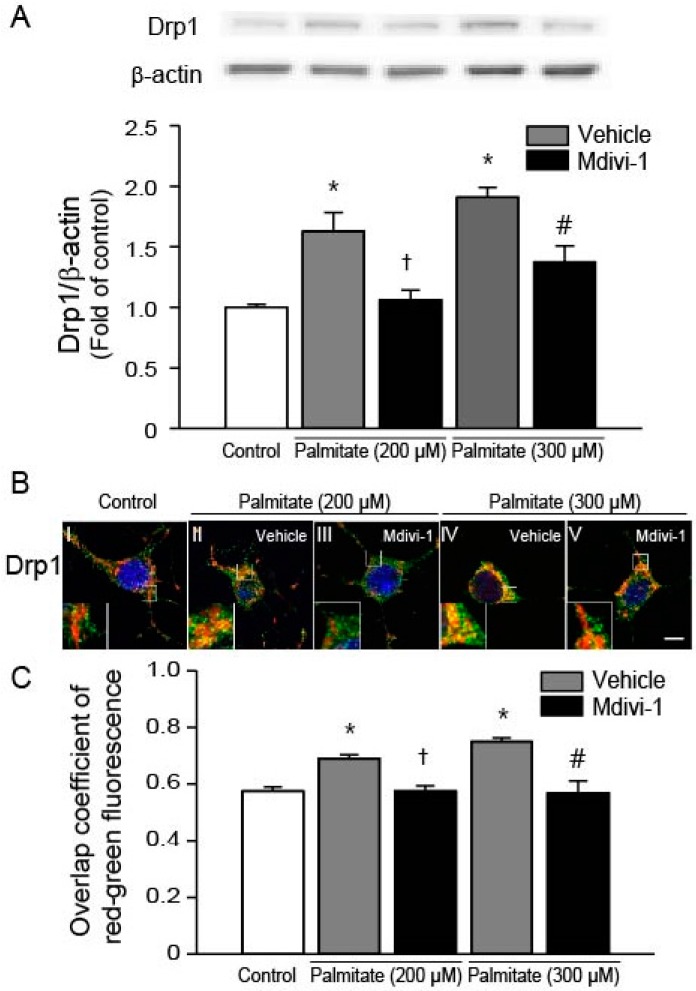
Mdivi-1 inhibits palmitate-induced Drp1 expression and mitochondrial translocation. (**A**) Drp1 protein levels were assessed by Western blot. Drp1 band intensity was normalized to β-actin intensity; (**B**) Effect of mdivi-1 on Drp1 translocation from the cytosol to the mitochondria. At the completion of palmitate exposure, cells were stained with MitoTracker^®^ Orange CMTMRos (red) and then probed with Drp1 antibody (green) to detect subcellular localization of Drp1. Nuclei were visualized by staining with DAPI. Representative microscopic images show immunofluorescent detection of Drp1. Scale bar = 5 μm; (**C**) Quantitative analysis for overlap coefficient of red-green fluorescence. Values are the mean ± SEM of four separate experiments performed in triplicate. * *p* < 0.05 vs. DMSO-treated control; † *p* < 0.05 vs. 200 μM palmitate; # *p* < 0.05 vs. 300 μM palmitate.

It has been shown that Drp1 is one of the key regulators of apoptosis [38] and palmitate induces expression of Drp1 [30,39]. Western blot analysis of Drp1 revealed that the expression levels of Drp1 were significantly increased in hippocampal NSCs exposed to palmitate, which were attenuated by the pretreatment of cells with mdivi-1 (Figure 2A). During apoptotic process, Drp1 translocates from the cytosol to the mitochondria [28]. To investigate the effect of palmitate on the subcellular localization of Drp1, we performed immunofluorescence confocal microscopy. As shown in Figure 2B, Drp1 (green) did not colocalize with red mitochondria (image I). The yellow merged color was enhanced when cells were exposed to palmitate (Figure 2B, images II and IV), indicating that Drp1 was translocated into the mitochondria from the cytosol. However, mdivi-1 treatment almost completely reversed palmitate-induced translocation of Drp1 (Figure 2B, images III and V). Quantitative analysis of colocalization of green Drp1 and red MitoTracker signals yielded overlap coefficients, as previously described by Manders et al. [40]. Our data revealed that overlap coefficient values were increased by palmitate, whereas mdivi-1 led to significant reduction of overlap coefficient (Figure 2C).

### 2.3. Mdivi-1 Inhibits Palmitate-Induced Increase in ROS Generation

**Figure 3:**
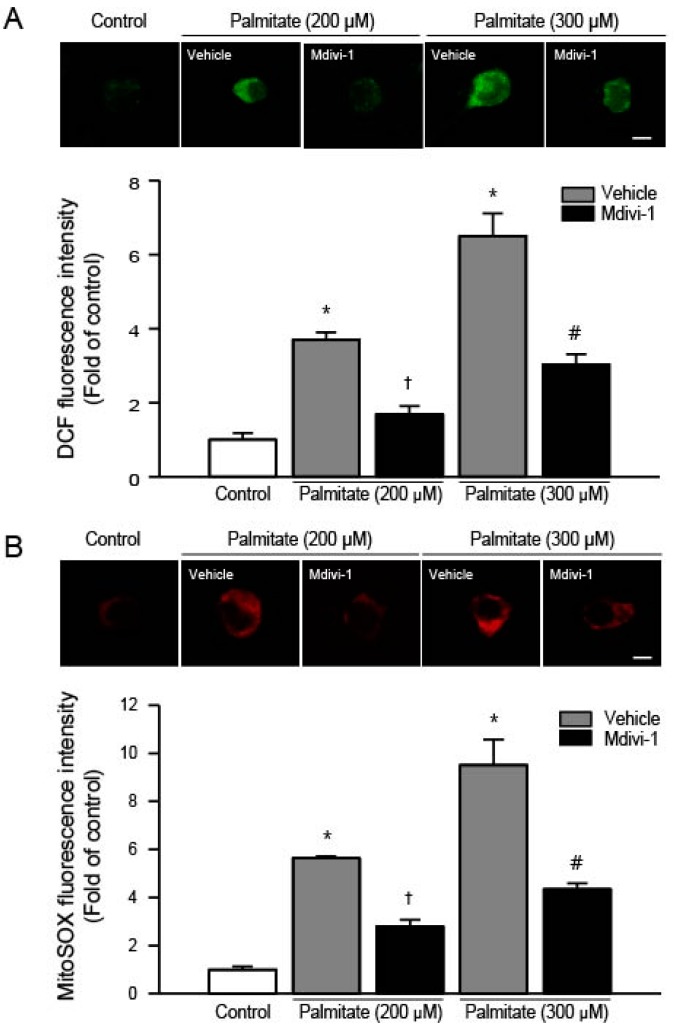
Effect of mdivi-1 on the intracellular and mitochondrial ROS production in hippocampal NSCs exposed to palmitate insult. Cells were pretreated with DMSO vehicle or mdivi-1 (20 μM) for 2 h and then exposed to 0.5% BSA vehicle or palmitate (200 and 300 μM) for 24 h. (**A**) Intracellular ROS levels were determined using confocal microscopy on cells stained with the ROS-sensitive fluorescent dye 2’,7’–dichlorofluorescin diacetate (DCFDA). Representative microscopic images show DCFDA-stained hippocampal NSCs (upper panel). Scale bar = 5 μm. Quantitative analysis (lower panel) showed that DCF fluorescence was increased by palmitate exposure, whereas mdivi-1 inhibited palmitate-induced increase in DCF fluorescence; (**B**) Mitochondrial ROS levels were determined using confocal microscopy on cells stained with the mitochondrial superoxide indicator MitoSOX Red. Representative microscopic images show MitoSOX Red-stained hippocampal NSCs (upper panel). Scale bar = 5 μm. Quantitative analysis (lower panel) showed that MitoSOX Red fluorescence was increased by palmitate exposure, whereas mdivi-1 inhibited palmitate-induced increase in MitoSOX Red fluorescence. Values are the mean ± SEM of four separate experiments performed in triplicate. * *p* < 0.05 vs. DMSO-treated control; † *p* < 0.05 vs. 200 μM palmitate; # *p* < 0.05 vs. 300 μM palmitate.

To determine whether mdivi-1 may inhibit palmitate-induced apoptotic cell death by suppression of ROS generation, we examined the changes in intracellular ROS production. Compared with vehicle-treated cells, hippocampal NSCs exposed to palmitate (200 and 300 μM) displayed increased ROS levels in a concentration-dependent manner (Figure 3A). In contrast, pretreatment of cells with mdivi-1 significantly reduced the increase in 2′,7′-dichlorodihydrofluorescein diacetate (DCFDA) fluorescence induced by palmitate (Figure 3A). Next, we also measured mitochondrial ROS production using MitoSOX Red, which is a redox-sensitive dye to mitochondria. It has been reported that most intracellular ROS are generated by mitochondria [41]. Palmitate exposure increased mitochondrial ROS levels in a concentration-dependent manner when compared to controls, whereas mdivi-1 significantly reduced the increase in MitoSOX Red fluorescence induced by palmitate (Figure 3B).

### 2.4. Mdivi-1 Stabilizes Mitochondrial Transmembrane Potential (Δψ_M_)

**Figure 4:**
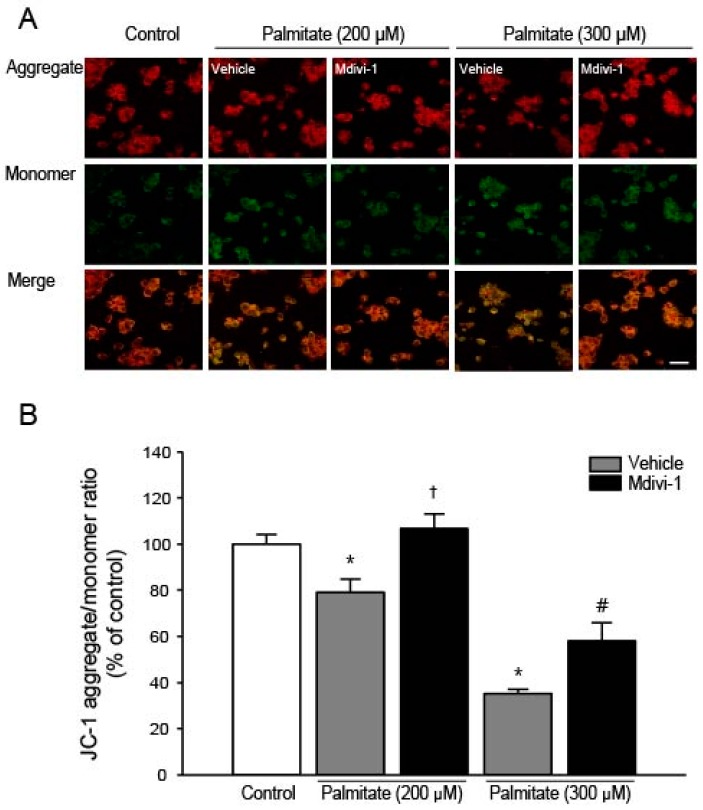
Effect of mdivi-1 on mitochondrial transmembrane potential in hippocampal NSCs exposed to palmitate insult. Cells were pretreated with DMSO vehicle or mdivi-1 (20 μM) for 2 h and then exposed to 0.5% BSA vehicle or palmitate (200 and 300 μM) for 24 h. (**A**) The mitochondrial transmembrane potential was determined by confocal microscopy using JC-1 dye. Mitochondria with a higher membrane potential have a red fluorescence, and those with a lower membrane potential are green. Scale bar = 20 μm; (**B**) The ratio of red-to-green fluorescence was quantified in cells of interest using the measurement functions in the Carl Zeiss LSM 700 Meta software. Values are the mean ± SEM expressed as percent of control. * *p* < 0.05 vs. DMSO-treated control; † *p* < 0.05 vs. 200 μM palmitate; # *p* < 0.05 vs. 300 μM palmitate.

It has been shown that palmitate destabilizes Δψ_M_ due to the opening of the mitochondrial permeability transition (PT) pore [42] and mdivi-1 ameliorates apoptosis by preventing mitochondrial depolarization [43]. Therefore, we examined the effect of mdivi-1 on Δψ_M_ using 5,5′,6,6′-tetrachloro-1,1′,3,3′-tetraethylbenzimidazolylcarbocyanine iodide (JC-1), which can be used as an indicator of Δψ_M_. JC-1 shows potential-dependent accumulation in energized mitochondria as indicated by a fluorescence shift from green (~525 nm) to red (~590 nm). At low concentrations (due to low Δψ_M_), JC-1 is predominantly a monomer that yields green fluorescence. At high concentrations (due to high Δψ_M_), the dye aggregates yielding a red fluorescence. The ratio between green and red depends on Δψ_M_, and mitochondrial depolarization is indicated by a decrease in the red/green fluorescence intensity ratio [44]. As shown in Figure 4A, it was found that Δψ_M_ was significantly decreased as indicated by the marked increases in green fluorescence when cells were exposed to increasing dose of palmitate. However, when cells were pretreated with mdivi-1, palmitate-induced mitochondrial depolarization was significantly attenuated (Figure 4B).

### 2.5. Mdivi-1 Increases Bcl-2 and Decreases Bax, Thereby Increasing the Bcl-2/Bax Ratio

**Figure 5:**
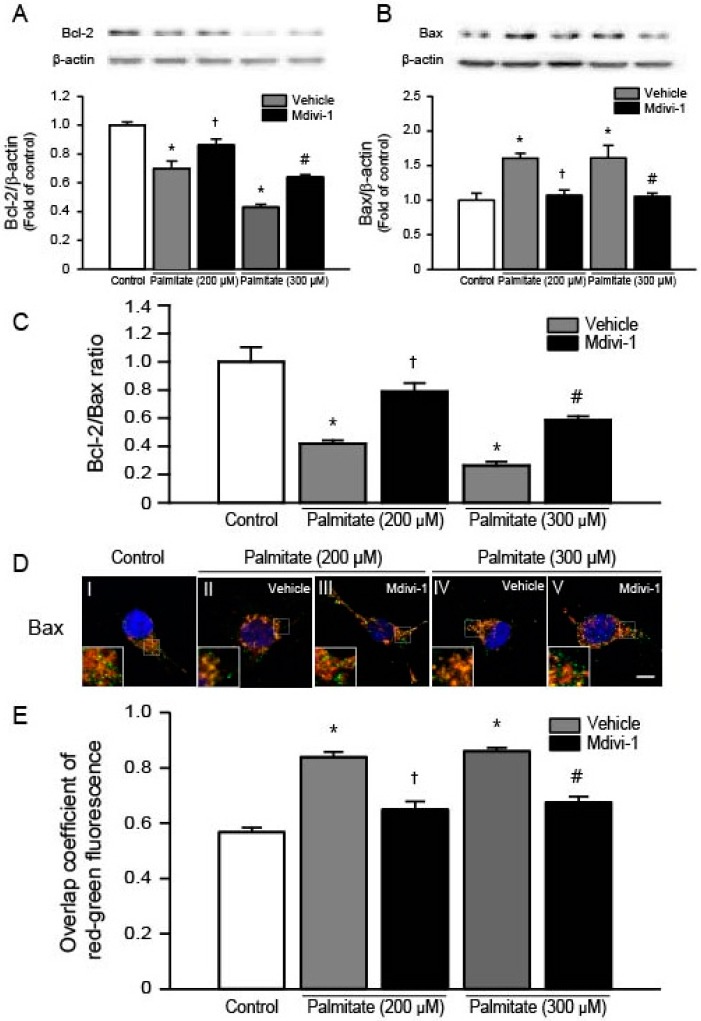
Effect of mdivi-1 on Bcl-2 and Bax levels in hippocampal NSCs exposed to palmitate insult. Cells were pretreated with DMSO vehicle or mdivi-1 (20 μM) for 2 h and then exposed to 0.5% BSA vehicle or palmitate (200 and 300 μM) for 24 h. Bcl-2 (**A**); and Bax (**B**) protein levels were assessed by Western blot. Bcl-2 and Bax band intensities were normalized to β-actin intensity; (**C**) Bar graphs show the ratio of protein levels of Bcl-2 to Bax; (**D**,**E**) Effect of mdivi-1 on Bax translocation from the cytosol to the mitochondria. At the completion of palmitate exposure, cells were stained with MitoTracker^®^ Orange CMTMRos (red) and then probed with Bax antibody (green) to detect subcellular localization of Bax. Nuclei were visualized by staining with DAPI. Representative microscopic images show immunofluorescent detection of Bax (**D**). Scale bar, 5 μm. Quantitative analysis for overlap coefficient of red-green fluorescence (**E**). Values are the mean ± SEM of four separate experiments performed in triplicate. * *p* < 0.05 vs. DMSO-treated control; † *p* < 0.05 vs. 200 μM palmitate; # *p* < 0.05 vs. 300 μM palmitate.

It has been shown that mdivi-1 inhibits apoptosis by upregulating the expression of anti-apoptotic protein Bcl-2 and downregulating pro-apoptotic protein Bax in primary hippocampal cells exposed to ischemia-reperfusion injury [35]. To examine changes in the protein levels of Bcl-2 and Bax, we performed Western blots using total lysates extracted from cells exposed to increasing dose of palmitate. We found that palmitate significantly decreased Bcl-2 protein levels in a dose-dependent manner (Figure 5A), whereas pretreatment of cells with mdivi-1 significantly increased Bcl-2 levels (Figure 5A). It was also found that Bax levels were significantly increased when cells were exposed to palmitate (Figure 5B). In contrast, mdivi-1 decreased Bax protein levels (Figure 5B), thereby significantly increasing the Bcl-2/Bax ratio (Figure 5C). Next, we conducted immunofluorescence staining with confocal microscopy to assess the precise subcellular localization of Bax. In vehicle-treated cells, Bax (green color) did not colocalize with mitochondria (red color) (Figure 5D, image I). The yellow merged color indicated the colocalization of Bax and mitochondria when cells were exposed to 200 μM (Figure 5D, image II) and 300 μM (Figure 5D, image IV) of palmitate, suggesting the translocation of Bax from the cytosol to the mitochondria after palmitate treatment. However, pretreatment of cells with mdivi-1 (Figure 5D, images III and V) decreased palmitate-induced translocation of Bax. In addition, quantitative colocalization analysis showed that overlap coefficient increased with palmitate, whereas mdivi-1 resulted in significantly decreased overlap coefficient values (Figure 5E).

### 2.6. Mdivi-1 Inhibits Palmitate-Induced Increase in Cytochrome C Release and Caspase-3 Activation

**Figure 6:**
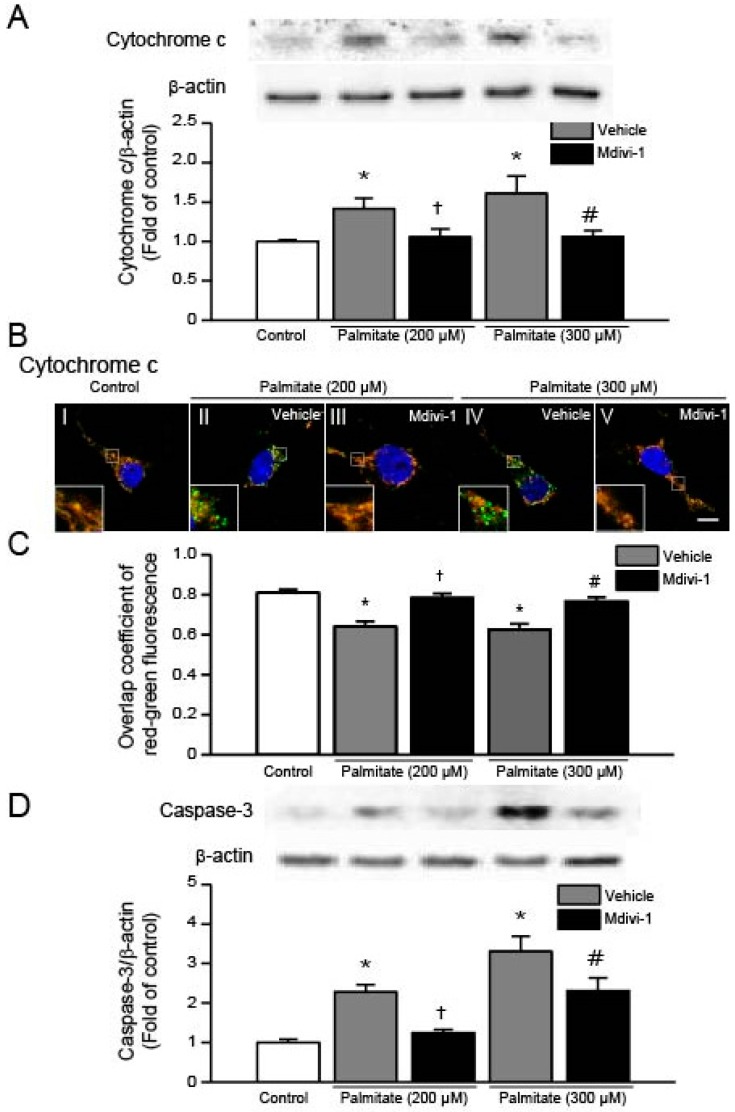
Effect of mdivi-1 on cytochrome c and caspase-3 levels in hippocampal NSCs exposed to palmitate insult. Cells were pretreated with DMSO vehicle or mdivi-1 (20 μM) for 2 h and then exposed to 0.5% BSA vehicle or palmitate (200 and 300 μM) for 24 h. Cytochrome c (**A**); and active caspase-3 (**D**) protein levels were assessed by Western blot. Cytochrome c and active caspase-3 band intensities were normalized to β-actin intensity; (**B**,**C**) Effect of mdivi-1 on cytochrome c translocation from the mitochondria to the cytosol. At the completion of palmitate exposure, cells were stained with MitoTracker^®^ Orange CMTMRos (red) and then probed with cytochrome c antibody (green) to detect subcellular localization of cytochrome c. Nuclei were visualized by staining with DAPI. Representative microscopic images show immunofluorescent detection of cytochrome c (**B**). Scale bar, 5 μm. Quantitative analysis for overlap coefficient of red-green fluorescence (**C**). Values are the mean ± SEM of four separate experiments performed in triplicate. * *p* < 0.05 vs. DMSO-treated control; † *p* < 0.05 vs. 200 μM palmitate; # *p* < 0.05 vs. 300 μM palmitate.

To investigate whether mdivi-1 inhibits palmitate-induced release of cytochrome c, we conducted Western blot analysis using lysates extracted from hippocampal NSCs. As shown in Figure 6A, we found a significant increase in cytochrome c protein levels when cells were exposed to palmitate. In contrast, pretreatment of cells with mdivi-1 resulted in a significant reduction of cytochrome c levels. We confirmed these results by immunofluorescence confocal microscopy and found that cytochrome c (green) was colocalized with MitoTracker (red) in vehicle-treated cells (Figure 6B, image I). It was shown that green fluorescence (cytochrome c) was remarkably enhanced and mitochondria were not stained green for cytochrome c but stained with red MitoTracker (Figure 6B, images II and IV), indicating release of cytochrome c from the mitochondria into cytosol. In the meantime, mdivi-1 treatment decreased palmitate-induced increment in green fluorescence and recovered colocalization of cytochrome c and MitoTracker staining (Figure 6B, images III and V). In addition, to quantify for colocalization of cytochrome c and mitochondria, we calculated the overlap coefficient (Figure 6C). Next, we examined whether palmitate induces activation of caspase-3 in hippocampal NSCs and found that palmitate exposure resulted in increase in protein levels of active caspase-3 (Figure 6D). By contrast, mdivi-1 significantly reduced palmitate-induced augmentation in active caspase-3 protein levels (Figure 6D).

## 3. Discussion

Mdivi-1, a small molecule selective inhibitor of the self-assembly of Drp1, is known to prevent mitochondrial fission and protects cells against apoptotic cell death [30,32,33,34,35,36,45,46]. In this study, our data demonstrate that mitochondrial division inhibitor mdivi-1 protects hippocampal NSCs against palmitate-induced lipotoxicity. We provided evidence that mdivi-1 suppresses intracellular and mitochondrial ROS generation and inhibits mitochondrial depolarization induced by high palmitate. We also demonstrate that mdivi-1 treatment changes the status of the Bcl-2 family proteins, inhibiting cytochrome c release and caspase-3 activation, thereby contributing to the promotion of survival of hippocampal NSCs exposed to palmitate.

It has been shown that mitochondrial fission is mediated by Drp1, a large cytosolic dynamin-related GTPase, that interacts with adaptor proteins on the mitochondrial membrane upon activation [28,47]. We found in this study that palmitate enhanced the expression of the mitochondrial fission protein Drp1, in agreement with previous reports [30,31,39], whereas the effects of palmitate on Drp1 levels could be prevented by pretreatment of cells with mitochondrial fission inhibitor mdivi-1. Drp1 is mainly located in the cytosol, but can be translocated to the mitochondria upon apoptotic stimulation of cells [28] and this step is requisite to inducing mitochondrial fission [47]. Indeed, we observed the translocation of Drp1 from the cytosol to the mitochondria when cells were exposed to palmitate. To the best of our knowledge, this is the first report showing that palmitate induces transport of cytosolic Drp1 into the mitochondria in hippocampal NSCs. Palmitate-induced Drp1 translocation was also attenuated by mdivi-1 pretreatment. Similar findings were reported by Xie et al. [34], where mdivi-1 suppressed mitochondrial Drp1 protein levels in a model of acquired epilepsy in vitro. Collectively, our data suggest that mdivi-1 attenuates palmitate-induced apoptosis via decreasing the activity of Drp1 and the translocation of cytoplasmic Drp1 into the mitochondria.

Increased production of cytosolic ROS is known to play an essential role in apoptosis after palmitate exposure [48]. In agreement with this finding, we observed that palmitate insult significantly increased intracellular ROS levels in hippocampal NSCs. Since mitochondria are an important source of intracellular ROS [41,49] and increases in mitochondrial ROS generation induce cellular oxidative damage and tissue dysfunction [50], we therefore examined if palmitate exposure increased mitochondrial ROS levels and found that MitoSOX fluorescence intensity was significantly increased during palmitate exposure. It was reported that mdivi-1 effectively ameliorated palmitate-induced ROS production in C2C12 cells [30], suggesting that mdivi-1 may have antioxidant properties in palmitate-exposed hippocampal NSCs. To determine whether mdivi-1 may suppress palmitate-induced oxidative stress, we assessed changes in intracellular and mitochondrial ROS levels. Treatment of cells with mdivi-1 significantly inhibited the increases in DCFDA and MitoSOX fluorescence intensity induced by palmitate. Therefore, given the important role of lipotoxicity-mediated oxidative stress in the development and progression of diabetic complications [51], the ability of this chemical Drp1 inhibitor to suppress ROS production appears to be important for its protective mechanisms against palmitate insult. This notion was supported by the finding that Drp1-suppression by siRNA reduced mitochondrial ROS levels in the palmitate-treated myocytes [29]. The Bcl-2 protein is a reasonable target for the putative antioxidant potential of mdivi-1 because this protein has been implicated in the regulation of mitochondrial ROS by its effect on mitochondrial complex IV activity [52]. Therefore, we consider that the increased expression of Bcl-2 protein in mdivi-1-treated hippocampal NSCs is associated with the promotion of cell survival and the protection against palmitate-induced oxidative stress.

During palmitate overload, lipotoxicity-induced oxidative stress facilitates opening of PT pore, followed by release of cytochrome c from mitochondrial inter-membrane space and apoptotic cell death [48]. The PT pore is regulated by the Bcl-2 family proteins, such as Bcl-2 and Bax [52,53,54]. The anti-apoptotic protein Bcl-2 prevents apoptosis by maintaining the mitochondrial transmembrane potential and blocking the release of cytochrome c, whereas the pro-apoptotic protein Bax stimulates apoptosis by collapsing the mitochondrial membrane potential, leading to increased cytochrome c release. Indeed, we found that palmitate caused a significant decrease in the level of Bcl-2 and a significant increase in the level of Bax. In contrast, mdivi-1 increased Bcl-2 and decreased Bax levels in palmitate-treated hippocampal NSCs. In this study, we also observed that pharmacological inhibition of Drp1 prevented palmitate-induced mitochondrial membrane depolarization, suggesting that mdivi-1 may inhibit mitochondrial PT pore opening in hippocampal NSCs exposed to palmitate. Taken together, these results suggest that this chemical inhibitor of Drp1 stabilizes the mitochondrial membrane potential by regulating Bcl-2 family proteins during palmitate exposure.

Although the exact molecular mechanisms of mdivi-1 inhibition of apoptosis during palmitate insult remain unclear, the Bcl-2 family proteins may be involved in the protective mechanism of mdivi-1. It is considered that the intracellular ratio of Bcl-2 to Bax acts as a predictor that determines the fate of cells [55], therefore, the ability of mdivi-1 in complete restoration of the Bcl-2/Bax ratio to normal levels in palmitate-treated hippocampal NSCs appears to contribute to the promotion of cell survival and the attenuation of the mitochondrial apoptotic pathway. Similar findings were observed in primary hippocampal cells, in which mdivi-1 protects cells against ischemia-reperfusion injury via upregulating Bcl-2 expression and downregulating Bax expression, thus increasing the ratio of Bcl-2/Bax [33,35]. Bax is mainly located in the cytosol under normal conditions but activated Bax is translocated to the mitochondrial outer membrane in response to apoptotic stimuli, such as palmitate [56]. To determine the changes in the subcellular localization of Bax, we performed an immunocytochemical staining of Bax. We found that Bax was moved from the cytosol to the mitochondria by palmitate insult while mdivi-1 treatment completely restored the palmitate-induced translocation of Bax. Bcl-2 proteins inhibits Bax translocation and mitochondrial membrane permeabilization [54,57]. Therefore, palmitate-induced decrease in Bcl-2/Bax ratio may contribute to the activation of mitochondrial PT pore opening, followed by release of cytochrome c into the cytosol. In this study, translocation of mitochondrial cytochrome c into the cytosol was observed when cells were exposed to palmitate, as previously reported [58]. Apoptosis can be blocked by inhibiting cytochrome c release because transport of cytochrome c to the cytosol is required for the induction of mitochondrial apoptotic cascades [53,54]. Once released, cytochrome c forms the apoptosome, a complex composed of apoptosis-activating factor Apaf-1, procaspase-9 and adenosine triphosphate, leading to activation of caspase-9 and caspase-3, and resulting in apoptosis [53,54]. It has been reported that mdivi-1 exerts its anti-apoptotic effects on hippocampal cells by preventing cytochrome c release and inhibiting caspase-3 activation [45,46,59]. In agreement with these findings, in the current study, mdivi-1 prevented the palmitate-induced release of cytochrome c and subsequent activation of caspase-3, thus inhibiting activation of the apoptotic pathway.

In the current study, we found that the Drp1 inhibitor mdivi-1 did not completely restore the cell viability and mitochondrial function in hippocampal NSCs exposed to palmitate lipotoxicity. These findings suggest that Drp1 may not be the major target during palmitate-mediated cell death. Palmitate is known to induce the disturbance of intracellular Ca^2+^ homeostasis [48]. Palmitate-induced oxidative stress enhances the release of Ca^2+^ from the endoplasmic reticulum, leading to overload of Ca^2+^ in cytosol and mitochondria. Excessive accumulation of Ca^2+^ in mitochondria may further aggravate mitochondrial ROS production and promote PT pore opening, which may lead to release of cytochrome c and provocation of caspase activation and cell death. Although it has been reported that mdivi-1 reduces cytosolic Ca^2+^ levels in myocardial ischemia-reperfusion injury [60], it remains to be determined whether mdivi-1 can attenuate palmitate-induced intracellular calcium dysregulation in hippocampal NSCs.

## 4. Materials and Methods

### 4.1. Materials

Mdivi-1 and sodium palmitate were obtained from Sigma-Aldrich (St. Louis, MO, USA). Neural stem cell culture media, Dulbecco Modified Eagle Medium (DMEM)/F12, and B27 supplement were from Gibco/Invitrogen (Carlsbad, CA, USA). All tissue culture reagents were obtained from Gibco/Invitrogen, and all other reagents were obtained from Sigma-Aldrich unless otherwise indicated.

### 4.2. Adult Rat Hippocampal NSCs Cultures and Treatment

Adult rat hippocampal NSCs were purchased from Chemicon^®^ (Catalog No. SCR022, Billerica, MA, USA) and cultured as previously described [15]. Cells used in this study are primary NSCs isolated from the hippocampus of adult fisher 344 rats. These cells were grown in DMEM/F12 containing l-glutamine, B27 supplement, 1X solution of penicillin/streptomycin, and basic fibroblast growth factor. Cells were plated on tissue culture plates that coated with poly-l-ornithine (10 μg/mL) and laminin (5 μg/mL). The hippocampal NSCs were maintained at 37 °C in 5% CO_2_ humidified incubator and passaged once every 5–6 days. Sodium palmitate solutions were prepared as previously described [61]. Briefly, sodium palmitate was fully dissolved in 0.1 M NaOH in 70 °C water bath. A 5% (*w*/*v*) solution of FFA-free bovine serum albumin (BSA)/PBS was prepared. Then, a 5 mM palmitate/BSA solution was prepared by appropriate mixture of the two solutions mentioned above. Finally, the mixture was readily further diluted in DMEM/F12 to obtain the desired final concentrations. To determine the effect of palmitate, cells were treated with vehicle (0.5% fatty free BSA) or 0.5% BSA conjugated-palmitate (200 or 300 μM) for 24 h. To assess whether mdivi-1 protects rat hippocampal NSCs from palmitate insult, cells were pretreated with DMSO vehicle or mdivi-1 (20 μM) for 2 h. This dose was selected on the basis of previous report showing that it had no effect on survival of neuronal cells [37].

### 4.3. Detection of Cell Death and Apoptosis

Cell viability was assessed by the MTT assay. To measure fragmented DNA, TUNEL staining was carried out using the ApopTag^®^ Fluorescein In Situ Apoptosis Detection kit (Chemicon, Temecula, CA, USA) according to the manufacturer’s instructions. Cells were counterstained with 4-6-diamidino-2-phenylindole (DAPI) before mounting.

### 4.4. Detection of Intracellular and Mitochondrial ROS Production

Intracellular ROS levels were assessed using the ROS-sensitive fluorescent dye DCFDA (Sigma-Aldrich). The cells were incubated with 2.5 μM DCFDA for 15 min. For the detection of mitochondrial ROS production, we used the mitochondrial-sensitive dye MitoSOX Red (Molecular Probes, Eugene, OR, USA). Hippocampal NSCs were incubated with 3 μM MitoSOX Red for 10 min. Fluorescence was captured using a 40× objective lens on a Carl Zeiss LSM 700 Meta confocal microscope (485-nm excitation and 535-nm emission for DCFDA; 510-nm excitation and 580-nm emission for MitoSOX Red). DCFDA and MitoSOX Red fluorescences were quantified from cells of interest using the measurement functions on the Carl Zeiss confocal software.

### 4.5. Assessment of Mitochondrial Membrane Potential (Δψ_M_)

Mitochondrial membrane potential was assessed with the fluorescent dye JC-1 Mitochondrial Membrane Potential Detection kit (Stratagene, La Jolla, CA, USA) and confocal microscopy according to the manufacturer’s instructions. In brief, cells were incubated with 1× JC-1 reagent solution at 37 °C for 15 min. Culture slides were washed and mounted with PBS, and confocal images were acquired by the Carl Zeiss LSM 700 Meta confocal microscope. The red-to-green fluorescence ratio was quantified from cells of interest using the measurement functions on the confocal microscopy software.

### 4.6. Western Blot Analysis

For Western blot analysis, cells were lysed in a buffer containing 20 mM Tris–HCl (pH 7.4), 1 mM EDTA, 140 mM NaCl, 1% (*w*/*v*) *Nonidet* P-40, 1 mM Na_3_VO_4_, 1 mM phenylmethylsulfonyl fluoride, 50 mM NaF, and 10 μg/ml aprotinin. Cell lysates were separated by 15% SDS-PAGE and electrotransferred onto polyvinylidene difluoride membranes (Bio-Rad, Hercules, CA, USA). The membranes were soaked in blocking buffer (1× Tris-buffered saline, 1% BSA, 1% nonfat dry milk) for 1 h and incubated overnight at 4 °C with the primary antibodies against Drp1 and Bax (Abcam, Cambridge, UK; 1:1000), active caspase-3, Bcl-2, and cytochrome c (Cell signaling, Danvers, MA, USA), and β-actin (Santa Cruz Biotechnology, Santa Cruz, CA, USA). All blots were developed using a peroxidase-conjugated anti-rabbit IgG and a chemiluminescent detection system (Santa Cruz Biotechnology). The bands were visualized using a ChemicDoc XRS system (Bio-Rad, Hercules, CA, USA) and quantified using Quantity One imaging software (Bio-Rad).

### 4.7. Immunocytochemical Detection of Drp1 or Bax Translocation and Cytochrome C Release

At the completion of palmitate exposure, hippocampal NSCs were stained with MitoTracker^®^ Orange CMTMRos (200 nM) for 10 min at 37 °C and then fixed with 4% PFA. Cells were blocked for 1 h with 1% BSA and 0.3% normal goat serum in PBS and incubated with primary antibodies against Drp1, Bax (1:1000, Abcam) or cytochrome c (1:1000, Santa-Cruz Biotechnology) overnight at 4 °C. Then, cells were incubated with Alexa Fluor 488-conjugated secondary antibody against rabbit IgG for 2 h at room temperature. The images were captured by the Carl Zeiss LSM 700 Meta confocal microscope. Overlap coefficient was measured using the measurement functions on the Carl Zeiss confocal software (Zen 2009 SP2, Carl Zeiss Microscopy GmbH, Jenna, Germany).

### 4.8. Statistical Analysis

Data are presented as mean ± SEM of four different experiments (each experiment was performed in triplicate). Statistical analysis between groups was performed using one-way ANOVA and Holm–Sidak method for multiple comparisons using SigmaStat for Windows Version 3.10 (Systat Software, Inc. Point Richmond, CA, USA). *p* < 0.05 was considered statistically significant.

## 5. Conclusions

In conclusion, our data indicate that attenuating palmitate-induced expression and mitochondrial translocation of Drp1 may serve as a novel therapeutic target for the preservation of mitochondrial function, thereby inhibiting oxidative stress-induced apoptosis. Inhibition of palmitate-induced oxidative stress and improvement of mitochondrial impairment by mdivi-1 may be considered as valuable therapeutic strategy for the treatment of diabetes-associated cognitive dysfunction.

## Abbreviations

NSCs	Neural stem cells
ROS	Reactive oxygen species
FFA	Free fatty acid
Drp1	Dynamin-regulated protein 1
BSA	Bovine serum albumin
DCFDA	2′,7′-Dichlorodihydrofluorescein diacetate
JC-1	5,5′,6,6′-Tetrachloro-1,1′,3′,3′-tetraethylbenzimidazolylcarbocyanine iodide
